# Explaining Support for COVID-19 Cell Phone Contact Tracing

**DOI:** 10.1017/S0008423921000019

**Published:** 2021-01-14

**Authors:** Ludovic Rheault, Andreea Musulan

**Affiliations:** 1 Department of Political Science and Munk School of Global Affairs and Public Policy, University of Toronto, 100 St George Street, Sidney Smith Hall 3018, Toronto, ON, M5S 3G3; 2 Department of Political Science, University of Toronto, 100 St George Street, Sidney Smith Hall 3018, Toronto, ON, M5S 3G3

**Keywords:** COVID-19, cell phone contact tracing, public opinion, disease avoidance, survey experiment, framing, COVID-19, applications de traçage des contacts, opinion publique, psychologie évolutionniste, méthode expérimentale, cadrage cognitif

## Abstract

Contact tracing applications have been deployed at a fast pace around the world to stop the spread of COVID-19 and may be key to containing future pandemics. This study aims to explain public opinion toward cell phone contact tracing using a survey experiment. We build upon a theory in evolutionary psychology—disease avoidance—to predict how media coverage of the pandemic affects public support for containment measures. We report three key findings. First, exposure to a news item that shows people ignoring social distancing rules causes an increase in support for cell phone contact tracing. Second, pre-treatment covariates such as anxiety and a belief that other people are not following the rules rank among the strongest predictors of support for COVID-19 apps. And third, while a majority of respondents approve of the reliance on cell phone contact tracing, concerns for rights and freedoms remain a salient preoccupation.

## Résumé

Les applications de traçage des contacts ont été déployées à un rythme effréné dans plusieurs pays afin d'endiguer la propagation de la COVID-19, et pourraient devenir une technologie essentielle pour contrer de futures pandémies. Cette étude cherche à expliquer l'opinion publique face aux applications de traçage avec un devis expérimental administré par sondage. Nous invoquons une théorie en psychologie évolutionniste pour prédire comment la couverture médiatique de la pandémie affecte le soutien envers les politiques du government. Nous présentons trois conclusions. Premièrement, l'exposition à des reportages montrant des personnes qui ignorent les règles de distanciation sociale fait croître l'appui aux applications de traçage. Deuxièmement, le niveau d'anxiété et la croyance que les autres ne respectent pas les règles de distanciation figurent parmi les plus importants facteurs expliquant une opinion favorable aux applications de traçage. Troisièmement, même si une majorité de répondants approuvent les applications de traçage, les préoccupations par rapport aux droits et libertés sont répandues.

## Introduction

Containing a pandemic like the coronavirus has brought the state back into the daily life of citizens, to an extent arguably not seen in decades. The government response entails a trade-off that is fundamental to political science: the extent to which people are willing to relinquish their civil liberties for the benefit of society.^1^ Cell phone contact tracing apps—applications designed to facilitate the process of contact tracing—encapsulate such a trade-off. These apps have the potential to protect the public while avoiding the need for large-scale restrictions on economic activities during a pandemic (Ferretti et al., 2020; Peak et al., 2020; WHO, 2020). However, they also involve design choices that may encroach on the right to privacy, if only by increasing the capacity of the state to collect information about individual health conditions (Bengio et al., 2020). Some countries have gone further by legally enforcing the usage of a COVID-19 app (see O'Neill et al., 2020). The COVID Alert app launched by the Canadian federal government in July of 2020 is voluntary and designed with privacy protection features. However, it attracted less than five million users in three months, or roughly 12.5 per cent of the population (Canada, 2020). Since the effectiveness of these apps depends on the rate of adoption (Braithwaite et al., 2020), understanding public opinion on this question is key for a successful implementation.

This research note's objective is to examine the effect of mass communication on public support for cell phone contact tracing. We draw from evolutionary psychology to explain attitudes toward policy responses in times of pandemic. We argue that media coverage emphasizing the risk-prone behaviour of people ignoring social distancing—a common occurrence during the COVID-19 pandemic—should increase support for a more stringent policy response. Conversely, we expect news items suggesting that a majority of the population will be infected to reduce support for containment measures that may encroach on privacy, by forcing the public to consider themselves as potential carriers. We test both hypotheses using a survey experiment conducted on a representative sample of Canadian respondents (*N* = 1,200), in which we randomly exposed participants to real-life news items. We also measured various attitudes using traditional survey questions, and we asked respondents to elaborate on their opinion about cell phone contact tracing specifically, using an open-ended question. Our results show that perceptions of other people flouting social distancing rules—both stimulated by the treatment and self-reported—are a key determinant of support for cell phone contact tracing.

COVID apps can facilitate the industrious process of contact tracing, one of the primary methods used by governments to contain the spread of viruses during an epidemic. On May 7, 2020, the *MIT Technology Review* identified 25 countries with active COVID-19 app campaigns, five of which were making the contact tracing app mandatory (O'Neill et al., 2020).^2^ Using either GPS geolocation or Bluetooth technology, these apps have the common characteristic of keeping a record of interactions between users who come in proximity to each other, for various periods of time. When a new case of COVID-19 is identified, users who have been in contact with the infected person are notified. Scholars and human rights advocates have emphasized various concerns about the implementation of cell phone contact tracing (Cho et al., 2020; Stanley and Granick, 2020; Kahn et al., 2020; Parker et al., 2020). Aside from security risks associated with the technology, COVID-19 apps may increase the effectiveness of authorities in gaining information about the health risks of specific individuals. A common design choice is to have the app inform health agencies of the identity of users who have been in contact with an infected individual, similar to manual contact tracing (see Cohen et al., 2020).^3^ The app used in Alberta during the COVID-19 pandemic (ABTraceTogether) is based on this approach: it shares the name and phone number of users exposed to the virus with provincial health authorities (Alberta, 2020). On the other hand, the nationwide COVID Alert application launched in Canada sends the alerts to users only.

The Canadian case is particularly interesting given the remarkable resilience of the population in dealing with the early stages of the COVID-19 pandemic. Previous research has shown that Canadians were overwhelmingly supportive of social distancing measures during the spring 2020 lockdown (Sevi et al., 2020; Merkley et al., 2020; Pickup et al., 2020), despite the presence of a rather extensive set of restrictive policies in urban areas (Armstrong and Lucas, 2020). However, we still have limited information on the public's perceptions of containment measures such as cell phone contact tracing. A survey commissioned by senators has shown Canadian respondents to be supportive of COVID-19 apps. The study found that 80 per cent of respondents “support the use of mobile device data by public health officials to notify those who have been close to someone who has tested positive for COVID-19” (Moodie et al., 2020: 20) and a majority of respondents (65%) supported the idea of a mandatory COVID-19 app. In contrast, a Mainstreet Research/iPolitics poll conducted shortly after using an interactive voice response (IVR) system found that a majority of respondents (57%) would consider it unacceptable if the government asked them to download a contact tracing app (Mainstreet Research, 2020). The present study helps to assess public opinion on this sensitive question and highlights factors that make people more or less likely to support cell phone contact tracing.

## Theory

Our objective is to explain how people cope with the trade-off between civil liberties and emergency measures to contain pandemics. Our argument stems from a well-established theory in evolutionary psychology, disease avoidance, which posits that humans have developed a natural response mechanism to avoid the threat posed by pathogens (Schaller, 2006). While political science research has relied on this theory to study attitudes toward outgroups such as immigrants and the homeless (Aarøe et al., 2017; Clifford and Piston, 2017), this response mechanism—also called the *behavioural immune system*—has an even more immediate relevance to understanding the formation of attitudes during a pandemic such as COVID-19. Due to evolutionary processes that have allowed human beings to survive through pandemics, we are predisposed to detect, and avoid, signs of infectious diseases (Schaller and Park, 2011). This explains, for instance, why many people feel disgust when seeing individuals with ostensible signs of infection (Aarøe et al., 2016). Faced with the spread of coronavirus, people will naturally seek clues that help them to identify the source of the threat and minimize the risks. As Lockyer and Hatemi (2014) pointed out, however, the presence of an evolutionary mechanism does not preclude individual variations in behaviours. The degree to which each person interprets the COVID-19 threat and the appropriate policy response may vary depending on the type of information they are exposed to, as well as other individual characteristics.

We argue that media coverage of the COVID-19 pandemic introduces frames that elicit predictable responses among the public. Framing, and more specifically emphasis framing, is a central theory in political communication, suggesting that the choice to emphasize a specific element of an issue may influence how people form opinions on that issue (Chong and Druckman, 2007, 2011; Cacciatore et al., 2016). For instance, the association between the virus and China in mass communications—one salient example was Donald Trump's discourse, who initially relied on the expression *Chinese virus*—may have pernicious effects, by presenting Chinese people as a potential vector of contagion.^4^ Individuals who incorporate such a frame may seek to avoid contact with Chinese-Americans, display hostility toward the group and demand a closing of the border for Chinese travellers. In fact, all of those behaviours have been observed in North America (see, for example, Tavernise and Oppel, 2020).^5^

In this study, we consider how emphasis frames used in media communication may affect public opinion toward containment measures specifically. We start from the general premise that COVID-19 has two distinctive features. First, there is a sense that the threat is real and important, reflected by the intensity of news coverage and by provinces declaring a state of emergency. Second, the low prevalence rate of confirmed cases makes the origin of threat particularly elusive.^6^ This context opens the door to multiple interpretations regarding who actually poses a contagion risk and what steps must be taken to avoid that threat.

We advance two hypotheses to explain how framing can influence public perceptions about the risk of contagion. First, news media frames emphasizing people who disregard social distancing guidelines should increase support for containment measures. A common emphasis frame during the pandemic was the coverage of people ignoring physical distancing rules.^7^ We expect this type of coverage to stress the idea that a specific group of the population—non-compliers—is a source of disease risk. The choice to emphasize non-compliance also suggests a causal interpretation that links a problematic behaviour with the spread of coronavirus. Consequently, we posit that people exposed to this frame are more likely to support tougher state interventions designed to contain the risk. A mandatory contact tracing application represents an intrusive type of state response to pandemics but one that may seem justified with the belief that negligent behaviour from other members of the public poses an increased risk of contagion. In summary, we expect that *news media coverage emphasizing non-compliers increases unconditional support for cell phone contact tracing* (Hypothesis 1).

Second, we expect another type of news coverage—one that emphasizes the idea that a virus is inevitable—to generate the opposite effect. We argue that exposing the public to the notion that COVID-19 will infect a majority of the population should reduce support for containment measures, especially mandatory cell phone contact tracing. News items of that nature were also common during the pandemic. For instance, an article circulated early on with projections that 30 to 70 per cent of Canadians would be infected with the virus (Dunham, 2020). This emphasis frame challenges the “optimism bias”—that is, the tendency that people have to believe they are unlikely to become infected themselves (see Van Bavel et al., 2020; Wise et al., 2020). Instead of depicting an external group of the population as a source of risk, this kind of information shifts the locus of the threat by forcing the public to consider a scenario in which they are potential carriers of the virus. As a result, we expect the consequences of government interventions to become salient and personal, in particular if a measure deals with health information. While the nationwide COVID alert application launched by the federal government in Canada ultimately came with some guarantees of anonymity, we expect that news coverage predicting a high rate of infection leads the public to become more cautious about embracing the idea of cell phone contact tracing. Thus, we expect that *news media coverage emphasizing that a majority of the population will become infected reduces unconditional support for cell phone contact tracing* (Hypothesis 2).

In short, these two frames suggest contrasting ideas to the audience: that the threat may originate from groups of reckless individuals, or that it may come from just about everyone. In the first case, the consequences of state interventions appear directed at external agents posing a risk of contagion. The second frame breaks the dissociation between the audience and the threat, with the consequence that state interventions appear directed at the audience itself.

## Data and Research Design

Our data come from an online survey of 1,200 Canadians recruited using the Cint platform, a market exchange for survey respondents. Coppock and McClellan (2019) provide a detailed examination of the validity of this type of survey platform for social science research. We fielded the survey in both official languages between May 28 and May 29, 2020. The survey used quota sampling based on census distributions for age, gender and region. We examined the quality of our sample by comparing proportions for additional demographic variables—income groups, education and ethnicity—against census proportions. Overall, the sample closely matches population distributions even for variables not utilized to establish the quotas. We report details of this analysis in the online Appendix.

The survey questionnaire contained five blocks of items presented on separate pages: (1) questions designed to measure pre-treatment covariates, (2) the randomized media treatment, (3) two questions measuring opinion on COVID-19 contact tracing applications, (4) questions on other government measures and (5) demographic questions.

We start by discussing the two questions designed to measure support for COVID-19 apps, the outcome variable of interest. All respondents were asked to answer a closed-ended query inviting them to indicate whether they support the government's participation in a COVID app. Next, all respondents were invited to elaborate on their opinion using an open-ended question. Our analysis focuses on both data types. Informed by previous surveys mentioned in the introduction, which led to divergent results, we paid particular attention to the drafting of the closed-ended question about COVID apps. We self-imposed the following criteria when designing that question: the nature of cell phone contact tracing must be described accurately using a simple language, the question should remain as neutral as possible (avoiding statements emphasizing the benefits of cell phone contact tracing over the risks, or vice versa) and the question should make clear that cell phone contact tracing involves the participation of governments. The baseline wording reads:

Many COVID-19 apps are being used around the world to notify people who were in contact with someone infected (contact tracing apps). These apps record the interactions between users by detecting when two cell phones are close to each other.

These apps require the participation of health agencies to confirm who tested positive for COVID-19.

Do you support the government's participation in a COVID-19 contact tracing app?

We offered three response categories: “Yes,” “Yes, but only if using the app is voluntary,” and “No.” This set of responses allows respondents to explicitly state whether their approval is conditional on the voluntary use of a contact tracing app.

To further validate measurement accuracy, we took two additional steps. First, we randomly assigned a total of three variations of the same question, to assess the sensitivity of the results to question wording. One alternative included the sentence “In most cases, COVID-19 apps are designed to notify health agencies when someone was in contact with an infected individual,” which captures an important design choice. For instance, the app used in Alberta was designed to share with provincial health authorities the contact information of persons who were near someone infected, as is the case with manual contact tracing. The other alternative mentioned both sides of the public debate regarding the use of contact tracing apps, with the sentence: “Some people claim that COVID-19 apps may pose a risk to fundamental rights, such as the right to privacy. Others claim these apps are needed to help reopen the economy while protecting public health.” These variations in question wording, however, have no statistically significant impact on the distribution of responses. We report a model that includes a direct test of the effect of question wording in the empirical section below.

Next, we asked all respondents to elaborate on their opinion using an open-ended question. The response rate was high: 90.7 per cent of respondents, or 1,088 out of 1,200, wrote a substantive answer. This number excludes gibberish text. The answers ranged from 1 to 139 words in length. These written comments provide an opportunity to conduct a more detailed analysis of public opinion. Responses were manually classified into a priori categories by three independent coders.^8^ The binary categories are not mutually exclusive and correspond to common arguments for and against cell phone contact tracing. Two of these arguments are in line with the theoretical mechanisms laid out in the previous section: whether people invoke the risk posed by other individuals to justify their position in support of COVID apps (Hypothesis 1) and whether people invoke the importance of restricting the scope of these apps (Hypothesis 2). We discuss the classification in detail and provide information on inter-rater reliability in the empirical section, whereas the full coding scheme appears in the online Appendix.

The experimental treatment in our survey consists of exposing respondents to a news item using one of the two frames discussed in the previous section. We randomly assigned respondents to three groups with equal probability. The first group was asked to read a news item describing people who neglect to abide by physical distancing rules in Toronto. The second group was asked to read a news item about the Canadian health minister indicating that 30 to 70 per cent of the population might become infected with COVID-19. The survey invited respondents to read an excerpt of the assigned news article, which included title, author, original image and the article lead (the vignettes appear in the online Appendix). Both news stories were published during the pandemic, and we selected them because a semantically equivalent French version also appeared in national media. The third group of respondents was not asked to read a news item and serves as a control.

A recent stream of literature has emphasized the role of attentiveness in survey experiments (Alvarez et al., 2019). While we acknowledge the importance of this question and considered the possibility of including an attention check in this study, we ultimately decided against it for a number of reasons. First, the reaction of respondents to attention checks has been debated and may introduce undesirable behaviours such as increased dropout (see, for example, Berinsky et al., 2016; Anduiza and Galais, 2017; Vannette and Krosnick, 2014). Second, our survey design is relatively simple and does not entail the kind of cognitive involvement required in more elaborate experiments (for instance, conjoint designs). Our reliance on large fonts and images in the media treatments is backed up by a literature showing that illustrations facilitate the processing of text (Glenberg and Langston, 1992), which simplifies the cognitive load for respondents. Finally, the open-ended question on COVID apps provides a way to monitor attention directly. We read each response individually, and we were able to verify that respondents who wrote valid comments understood the query. Furthermore, we replicated the results in the next section with and without respondents who did not answer the open-ended survey question. Omitting these respondents does not affect the main conclusions.

**Figure 1:**
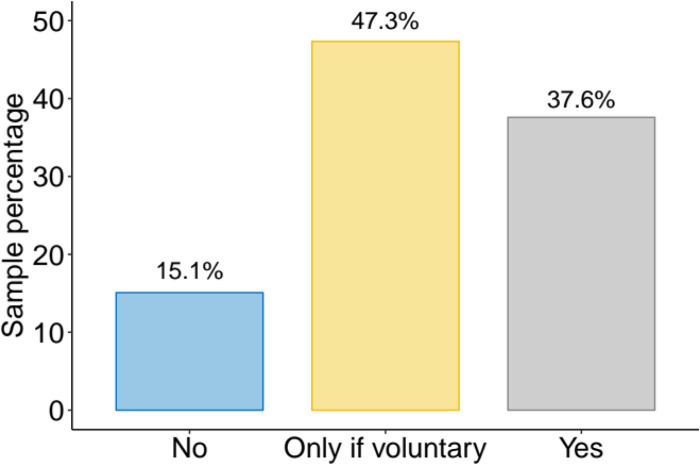
Distribution of Answers to COVID-19 Survey Question *Note:* The figure shows sample percentages across the three categories of the outcome variable, which are answers to a question asking “Do you support the government's participation in a COVID-19 contact tracing app?” Percentages are tabulated using the full sample, comprising all treatment groups (*N* *=* 1,200).

Figure 1 shows percentages of responses to the closed-ended survey question on COVID-19 cell phone contact tracing, our outcome variable of interest. For simplicity, we pool responses across the three variations of the question. A plurality of respondents endorsed the use of a COVID-19 app, but their support was conditional on participation being voluntary (47%). When combining with respondents who expressed unconditional support for COVID apps, the proportion favourable reaches 85 per cent. This is consistent with the high levels of support found in the Senate study (Moodie et al., 2020). Bengio et al. (2020) suggest that 56 per cent of the population must adopt a contact tracing app for it to be successful at abating the spread of coronavirus. Thus, based on the data observed in this survey, the successful deployment of a COVID app largely depends on convincing those whose support was only conditional.

For the analysis that follows, we focus on explaining unconditional support for COVID apps: those respondents who answered with a plain “Yes,” endorsing even the idea of a mandated COVID-19 contact tracing app. From a theoretical standpoint, this response category is the most interesting because it captures citizens willing to accept restrictions on liberties in response to the pandemic. We combine the categories “Only if voluntary” and “No” to create a binary dependent variable that equals 1 if the respondents answered “Yes,” and 0 otherwise. To further assess the plausibility of this decision, we fitted multinomial models with the three response categories and tested whether the “Only if voluntary” and “No” categories can be combined using a Wald test of the null that coefficients are equal across equations (Long, 1997: 162–63). We cannot reject the null at conventional levels of statistical significance, which supports the choice to combine these two response categories. Moreover, we note that the substantive conclusions we report below hold when considering a model that compares respondents who answered “Yes” versus “Only if voluntary”—the two most frequent answers.

## Results

**Figure 2:**
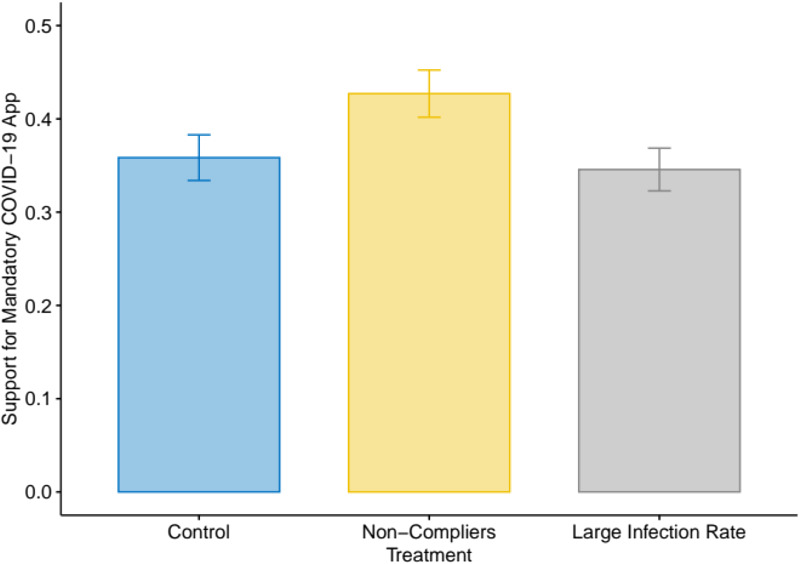
Support for Mandatory COVID-19 Apps, by Treatment Group *Note:* The figure shows the percentage answering an unconditional “Yes” to the closed-ended question on cell phone contact tracing across the three treatment groups, for the full sample (*N* *=* 1,200).

We begin by reporting the breakdown of our outcome variable across treatment groups. Figure 2 shows the proportion of respondents fully supporting cell phone contact tracing for each condition. We label “Non-Compliers” the first media treatment, which featured people not complying with social distancing guidelines. The second media treatment is labelled “Large Infection Rate.” The differences observed in Figure 2 are consistent with expectations from theory. Respondents presented with the Non-Compliers media frame are more likely to express an unconditional support for mandatory cell phone contact tracing. Conversely, those exposed to the idea that most of the Canadian population will be infected are less likely to do so. The first result is the most robust, as we detail below.

**Figure 3:**
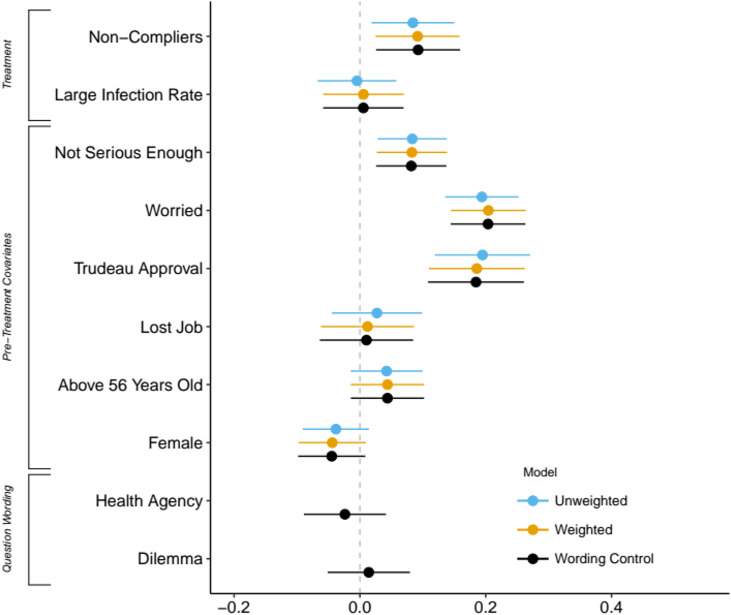
Support for Mandatory COVID-19 Apps (Average Treatment Effects) *Note:* The figure reports differences in predicted probabilities for a change from 0 to 1 in each predictor, along with 95 per cent confidence intervals, computed from logistic regression models. The raw output appears in the online Appendix. The dependent variable equals 1 if the respondent supports COVID-19 apps unconditionally, 0 otherwise. The “Unweighted” model does not include sampling weights. The “Weighted” model includes raking weights. The “Wording Control” model is computed with raking weights and includes dummy variables for variants of the COVID-19 apps question wording.

For a more accurate assessment, we report sample average treatment effects computed with confidence intervals in Figure 3. These estimates represent differences in predicted probabilities from logistic regression models that also include covariates and demographic variables (see online Appendix for the full tables and covariate definitions). The table shows results with and without raking weights, and the third model includes the wording used for the outcome variable as a predictor. Overall, we find that the Non-Compliers media frame increases support for mandatory cell phone contact tracing by about nine percentage points (everything else equal), a result that is statistically significant at the 95 per cent confidence level. This effect size is non-trivial. Combined with the support from theory, the reliance on randomization means that we can more safely interpret this effect in terms of causality. In contrast, the treatment effect for the second media frame under consideration is not robust. Our research design aimed to maximize external validity by relying on real-life news media articles. It is possible that the statement featured in the second treatment was not forceful enough to induce a change in attitudes, which could be explored in future research.

We can further validate the plausibility of our first hypothesis using traditional survey questions (pre-treatment covariates). A pre-existing belief that others are not taking physical distancing seriously is positively associated with support for COVID-19 apps (the “Not Serious Enough” variable in Figure 3). This relationship is consistent with the idea that people are more likely to seek remedial measures from the government when they perceive a group that poses a health risk. Controlling for this belief also accounts for a potential prior exposure to news items similar to the first media treatment. Next, we find that the level of anxiety regarding the virus matters. Respondents who declare they are “very worried” about their family members being infected by the virus are much more likely to support COVID-19 apps. This last result is particularly robust across the specifications considered.

Finally, we turn our attention to respondents’ written comments on COVID-19 apps. These comments are useful for understanding the considerations people have in mind when thinking about this technology. Immediately after asking respondents about COVID-19 apps, our survey invited them to elaborate on their opinion. The open-ended question began: “We would like to understand public opinion about COVID-19 apps. Could you please give us the main reason for your previous answer, in one or two sentences . . . ” As mentioned above, three coders independently classified the arguments invoked by respondents using a manual coding scheme. Table 1 reports the proportion of all respondents mentioning each argument type. The categories are non-exclusive, binary indicators equalling 1 if the respondent mentions a given type of argument, and 0 otherwise.

**Table 1:** Most Frequent Arguments about COVID-19 Apps

Category	%	Arguments	Top unigrams	κ
Civil Liberties	32.3	Impact on privacy; Importance of rights and freedoms; Being tested is an individual responsibility, not the state's.	privacy, government, invasive, governments, intrusive	0.89
With Conditions	21.1	Usage must be voluntary; App must be used for this purpose alone; Only infected should be required to use.	forced, mandatory, voluntary, choice, choose	0.80
Not Going to Work	8.9	Not everyone has a phone; People can leave their phones at home; Other methods are more effective.	phone, cell, smartphone, cells, rely	0.80
Threat Is Not Real	1.4	Threat is exaggerated by the government/media.		0.85
Societal Concerns	18.2	Public health above other considerations; Need to protect the vulnerable; Need to take action; Need to reopen the economy.	safety, worth, safe, safer, important	0.67
Others as a Threat	13.4	Need to locate the infected; Need to avoid contact with the infected; People not respecting the rules pose a risk.	infected, avoid, rules, distancing, contacted	0.75
App Is Effective	14.8	App would make contact tracing easier; Successful in other countries; App would provide useful data.	helpful, easier, useful, contact, great	0.56

*Note:* The table reports a classification of argument types for the written answers to the open-ended question on COVID-19 apps. The response rate was 90.7 per cent (1,088 out of 1,200). A residual category, not shown here, contains all other arguments that did not fit the coding scheme (3.8% of respondents, or 45). The second column is the percentage of written answers containing each argument type, out of 1,200 respondents. Percentages do not sum to 100 since the categories are not exclusive. The last column shows Cohen's kappa coefficients averaged across pairs of coders.

We designed the coding scheme based on our theory and prior knowledge of public debates surrounding cell phone contact tracing, which are discussed in the literature cited in our introduction. We started by identifying four types of arguments against COVID apps. Concerns about infringements of rights and freedoms, including the risks for privacy, constitute the first of these categories (we call it Civil Liberties for short). The second argument type focuses on the need to restrict the scope of COVID apps (With Conditions). The third argument against COVID-19 apps concerns their limited effectiveness (Not Going to Work). A last category targets beliefs that the virus threat may be exaggerated by governments or the media (Threat Is Not Real). Next, we constructed three categories of arguments in support of COVID apps, based on which consideration is salient: society, the source of the threat or the app itself. The first positive category is meant to capture one side of the trade-off involved by containment measures—that is, whether respondents are explicitly mentioning the benefits for society as their justification for supporting COVID apps (Societal Concerns).^9^ The second category includes arguments in line with our theoretical model based on disease avoidance: whether the respondent explicitly mentions the risk posed by the infected or by people not respecting the rules (Others as a Threat). The last category includes arguments focusing on the technology (App Is Effective).

Table 1 reports additional information about each category of the coding scheme. The penultimate column displays the top five unigrams (single words) most strongly associated with each category, calculated using the in-sample coefficients from a support vector classifier. These top words give an overview of the substantive content of written answers invoking each argument type, and they help to support the construct validity of the coding scheme. The last column of Table 1 reports the Cohen's kappa (κ) inter-coder reliability coefficients by category, averaged over each pair of coders. Overall, the level of agreement between coders is very strong for negative arguments about COVID apps, with values equal to or above 0.80.^10^ Coders were not as consensual for positive arguments, but given the interpretative nature of the task, a value of 0.75 for the Others as a Threat category, for instance, is more than satisfactory. Classes with a lower reliability score (for instance, arguments about the effectiveness of contact tracing apps) are not used for inference in what follows.

By far, the most frequent argument invoked by respondents concerned civil liberties, in particular the impact of COVID apps on privacy (mentioned by 32.3% of the 1,200 respondents). The importance of keeping contact tracing apps voluntary was also a recurring concern; those arguments are included in the second category (With Conditions). While we expected conspiracy theories about the virus to be prevalent, very few respondents made explicit statements suggesting that the threat is exaggerated (1.4% of respondents only). Among the positive arguments, comments focusing on the benefits to society were the most frequent (18.2%). Many respondents explicitly justified their support of COVID apps by the need to track people who pose a risk of infection (13.4%), which we expect to be primed by the Non-Complier news media coverage. In fact, several respondents interpreted that contact tracing apps would allow them to locate and avoid the infected ahead of time, even though the COVID Alert app deployed in Canada does not provide that kind of information. Some respondents invoked combinations of arguments for and against COVID-19 apps (for example, a respondent supportive of COVID-19 apps explicitly acknowledging the risks for privacy). Nonetheless, each argument type is a very strong predictor of the discrete response categories discussed earlier.

**Figure 4:**
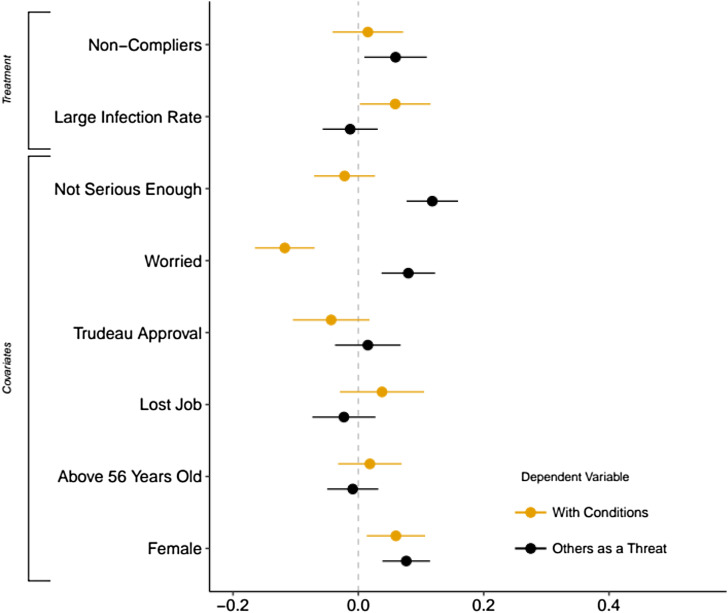
Determinants of Arguments on COVID-19 Apps (Average Treatment Effects) *Note:* The figure reports differences in predicted probabilities for a change from 0 to 1 in each predictor, along with 95 per cent confidence intervals, computed from logistic regression models. The full models appear in the online Appendix. The dependent variables equal 1 if the respondent invoked the argument type described in the legend, and 0 otherwise. The estimates are computed with raking weights.

This fine-grained categorization affords us with the opportunity to trace the causal mechanisms involved in the media treatment effects. Figure 4 reports results from logistic regression models similar to those used previously but using the type of argument mentioned by the respondent as outcome variables. For simplicity, we focus on the two noteworthy relationships. We find that exposure to the Non-Compliers news story is positively associated with arguments mentioning the risk posed by other individuals (Others as a Threat in Table 1). In turn, these considerations are positively related to unconditional support for COVID-19 apps (*p <* .001; test of difference in proportions). The self-reported belief that people are not serious enough is also a significant predictor of arguments invoking the risk posed by others. This gives credence to explanations drawn from disease avoidance theory. In contrast, exposure to the Large Infection Rate treatment is positively associated with arguments emphasizing the need to restrict the scope of the app (the With Conditions category). This time, the treatment effect is statistically significant at the 95 per cent confidence level. While the overall effect of the second media treatment did not seem robust, we can conjecture about the theoretical mechanism at work. People exposed to the idea that they could become infected themselves are more likely to consider the implications of a mandatory program and request the inclusion of safeguards. Future research would help to further assess the plausibility of such a mechanism.

## Conclusions

Our research design allowed us to examine the determinants of public support for containment measures during a pandemic, a topic for which there is still a paucity of research at the present time. We find that exposure to news items featuring people not respecting social distancing rules—a recurring type of emphasis frame during the COVID-19 pandemic—increases the level of support for contact tracing apps. The treatment is positively associated with explicit concerns about the risk posed by other individuals, and with support for mandatory cell phone contact tracing. This finding is consistent with expectations from disease avoidance theory, which posits that people confronted with an epidemic will naturally seek to identify the source of threat and look for solutions to abate the risk. We also expected that news coverage predicting a large infection rate would offset the tendency of people to dissociate themselves from the threat. We find weaker evidence of this type of effect. Nonetheless, respondents assigned to that treatment were more likely to mention the importance of restricting the scope of contact tracing apps.

Understanding the psychology of public opinion is key to predicting compliance with health safety measures during a pandemic. In particular, our results contribute to a literature focusing on the role of communication in the formation of attitudes toward health policy (see Lunz Trujillo et al., 2020). The reliance on fear appeals when communicating health risks to the public, for instance, is a well-documented strategy used to induce changes in behaviour (Witte and Allen, 2000; Van Bavel et al., 2020). Yet the way public officials and the media should communicate the threat during a pandemic remains an open question. Recent research has shown that news coverage during epidemics may also cause undesirable consequences such as hoarding and the burdening of medical facilities (McDonnell et al., 2012; Gollust et al., 2020; Garfin et al., 2020). Our findings suggest that media coverage of non-compliers during the COVID-19 pandemic led some respondents to endorse contact tracing apps, even if usage is mandated by the government. On the other hand, a danger with that communication practice is that it may encourage stigmatization, by spreading the belief that those infected with the virus are responsible for their fate. Respondents exposed to that treatment in our study were more likely to justify their support for COVID apps by emphasizing the need to locate the infected, as opposed to social benefits. Future research on the topic could greatly benefit the efficiency of communication strategies during health crises by revealing what types of attitudes the messages elicit.

The COVID-19 pandemic has had devastating economic consequences for Canadians, and the effect of the lockdown—one of the most extreme form of limitations to civil liberties—may be felt for a long time. Containment measures that may help to avoid the need for a full-scale lockdown, like cell phone contact tracing, will likely be on the agenda in the response to COVID-19 and future pandemics. In a democratic country, however, the success of automated contact tracing ultimately depends on public perceptions toward the technology. Our survey results illustrate the trade-off involved, with most respondents being ambivalent about COVID-19 apps. Some acknowledge the need to protect public health, but even more are concerned about implementation, privacy and freedom of choice. Addressing the contentious aspects of cell phone contact tracing effectively may be key for encouraging adoption during the fight against pandemics.

## Notes

1 For an overview of the pandemic's implications for rights and freedoms in Canada, see Macfarlane (2020).

2 Many more apps were actually deployed or in development as of May 2020. See, for example, https://benlevyx.github.io/covid-tracking/ for an overview.

3 This particular point can easily be a source of confusion. The Apple-Google exposure notification system used as a basis for many COVID apps was designed to notify health authorities when someone was in proximity to an infected individual, although specific implementations may diverge from that principle. As of June 18, 2020, the Apple-Google documentation stated that “no data will be shared by the system with public health authority apps unless one of the following two scenarios takes place,” with the second scenario being “if a user is notified through their app that they have come into contact with an individual who is positive for COVID-19” (Apple and Google, 2020: 5–6).

4 See also Motta et al. (2020) about the impact of media coverage of the pandemic and its effect on beliefs in conspiracy theories.

5 Previous research on political communication in Canada has also documented media framing on topics such as immigration and ethnicity (see, for example, Lawlor, 2015; Tolley, 2016).

6 As of January 2021, confirmed cases in Canada represented about 2 per cent of the population, using data from the Johns Hopkins Coronavirus Resource Center (Dong et al., 2020).

7 For example, our experimental treatment in this study uses a real news item covering a large gathering in downtown Toronto (Aguilar, 2020).

8 Two of the coders are authors, and the third was a student hired for this task. All English-language responses were classified by three coders, whereas the French-language responses were classified by two coders. We use the majority choice as our final response in the former case, and the few coder disagreements about French-language comments were manually resolved by the authors.

9 We initially devised a separate category for economic arguments—that is, the idea that cell phone contact tracing could avoid economic restrictions. However, this type of argument was seldom invoked by respondents. For simplicity, we combined this argument with the Societal Concerns category.

10 The average Cohen's kappa score for all seven categories combined is 0.78.

## Supplementary material

For supplementary material accompanying this paper visit https://doi.org/10.1017/S0008423921000019. click here to view supplementary material
