# Supplementary material for: Explaining Support for COVID-19 Cell Phone Contact Tracing
Source: Can J Polit Sci. 2021 Jan 14:1–18. doi: 10.1017/S0008423921000019 (PMC7925983; doi:10.1017/S0008423921000019)
Supplement: Supplementary file 1 [file S0008423921000019sup001.pdf]

Online Appendix
(Explaining Support for COVID-19 Cell Phone Contact Tracing)

Recruitment of Survey Respondents

Our internet survey responses were collected between May 28 and May 29 using the Cint platform. Recruitment relied on quota sampling—that is, we used target numbers matching the expected census distributions for age, gender, and region. Respondents from the territories were not included. The experimental protocol, including a statement of the theory and hypotheses, was reviewed and approved by the [blank] Research Ethics Board prior to launching the survey. The questionnaire was made available to respondents in both official languages, and the French version was written by a native speaker.

Opt-in, online surveys have become increasingly common for academic research with the decline in reliability of previous gold standards in public opinion research, e.g. random digit dialing. This mode of administration is even considered for major projects such as the Canadian Election Study (Breton et al., 2017). Previous research suggests that average treatment effects estimated from non-random surveys—the average impact of a randomized treatment, calculated on the sample—are reliable quantities of interest (Miratrix et al., 2018; Coppock and McClellan, 2019). Readers should remain wary of inferences involving population quantities, such as the percentage of the population supporting a given option.

To further assess data quality, we show in Table A1 that the sample provided by Cint is representative of the Canadian population on demographic variables other than the ones used for the quotas. We retrieved population proportions from the 2016 Census Profile data. While these data points were calculated four years ago, they provide a reasonably reliable benchmark to evaluate the sample. The representation of ethnic groups in the sample closely matches that in the population, with the exception of indigenous peoples. For most of the demographic groups listed in Table A1, the sample proportions could have been observed using probability sampling. All told, the quality of the sample is impressive, and provides a rather accurate representation of the Canadian population.

Table A1: Representativeness of Cint Sample

Demographic variable	Cint sample	2016 census	p-values
	Ethnicity		
Asian	0.177	0.164	0.323
Black	0.024	0.035	0.108
Hispanic or Latino	0.019	0.013	0.148
White	0.770	0.777	0.638
Indigenous	0.010	(0.024)	0.010*
	Education		
University degree	0.338	0.316	0.125
	Household Income		
\$0–\$14,999	0.074	0.059	0.055
\$15,000–\$24,999	0.095	0.090	0.593
\$25,000–\$49,999	0.225	0.247	0.111
\$50,000–\$79,999	0.242	0.249	0.613
\$80,000–\$99,999	0.154	0.118	<0.001*
\$100,000–\$149,999	0.145	0.155	0.421
\$150,000 or more	0.065	0.082	0.052

Notes: All census proportions come from the 2016 Canadian Census Profile tables. Proportions by ethnic group are from the item "Visible minority for the population in private households," with the exception of the "Registered and Treaty Indian" proportion used for the indigenous group, and marked in parentheses. The proportion with a university degree is for the Canadian population aged 25 and older. The household after-tax income is used for census income groups. The p-values are for Pearson chi-square tests of difference in proportions with Yates continuity correction. Significant differences indicate that the proportion is unlikely to be observed with random sampling.

$^* : p < 0.005$.

Questionnaire and Treatment Vignettes

The survey questionnaire presented to respondents contains five blocks. The first block included pre-treatment covariates (the variables labelled "Not Serious Enough" and "Worried" in the main text). The second block randomly assigned respondents to one of three groups and presented the media framing vignettes to the two treated subgroups. The third block queried respondents about their opinion toward cell phone contact tracing; the outcome variable of interest. The fourth block asked additional questions regarding containment measures. The fifth and final block contained demographic questions.

Figures A1 and A2 display the vignettes used for the two framing treatments administered during the survey. Each version invited respondents to read the excerpt from a news headline that included title, author, picture and lead. The two news articles were selected specifically because they captured the theoretical concept of interest, and since they had an equivalent version published in French media.

Figure A1: Media Frame Treatment I (Non-Compliers, English)

'It's selfish': Officials disappointed to see large crowds at downtown Toronto park amid pandemic

Bryann Aguilar, Published Saturday, May 23, 2020

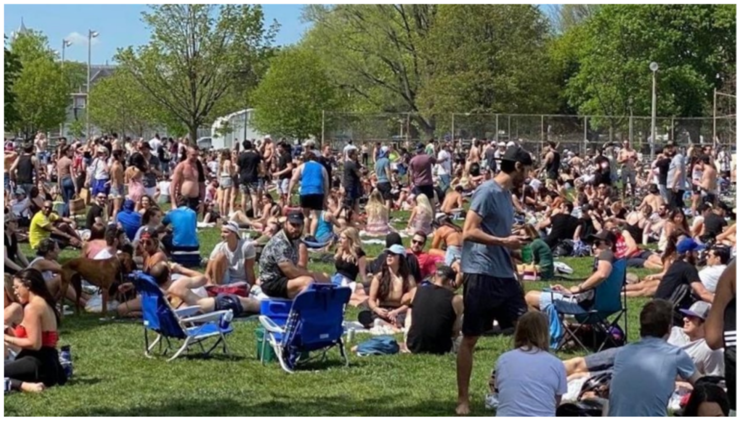

Toronto officials expressed their disappointment Saturday after large crowds gathered at a downtown Toronto Park to enjoy the summer-like weather appeared to have ignored physical distancing rules.

Thousands of people packed Trinity Bellwoods Park, located near Queen Street West and Ossington Avenue, despite repeated calls from public health officials to avoid large gatherings as Ontario continues to see a rise of new COVID-19 cases after being on the decline for weeks.

Figure A2: Media Frame Treatment II (Large Infection Rate, English)

COVID-19 could infect 30 to 70 per cent of Canadians: health minister

Jackie Dunham, Published Thursday, March 12, 2020

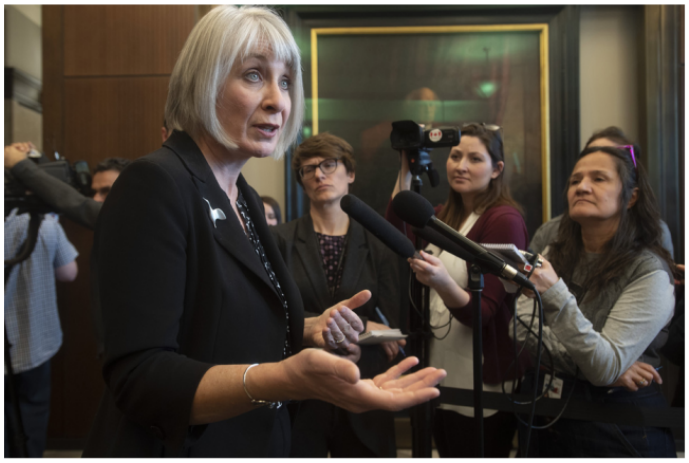

Between 30 to 70 per cent of the Canadian population could become infected with the novel coronavirus, according to federal Health Minister Patty Hajdu.

As explained in the manuscript, we devoted considerable time to ensure that the question used to ask respondents about COVID-19 apps remained as neutral as possible. One reason for this was the existence of conflicting results in previous polls. In the introduction to the main text, we mentioned the Senate study and the Mainstreet Research poll, two sources of data on public opinion on cell phone contact tracing that featured in Canadian news media. The phrasing of survey questions differed in these two studies, which raises the issue of how sensitive public opinion is to question wording. The Senate study's query contained a preamble explaining the purpose and benefits of contact tracing apps. While it provided respondents with background information about the technology, this choice may have painted the issue in a more positive light. The preamble and question read:

> Today's smart phones have location and proximity tracking capabilities. Used together with rapid testing capabilities, this technology could help public health professionals to more rapidly, accurately and completely trace the possible spread of COVID-19. This would allow them to protect public health and help to better manage the easing of social and economic restrictions.
>
> If the tracking capabilities of smart phones provided public health officials with the ability to anonymously and automatically notify all those who have been close to someone who tested positive for COVID-19, how supportive would you be of using this capability in Canada? (Moodie et al., 2020, 34).

The Mainstreet Research question, on the other hand, relied on a different language. It contained the verb "track", which may have primed the privacy implications of contact tracing apps. The question was:

> [...] Please tell us if you think it is acceptable or not: The government asking you to download an app on your smart-phone to track who you might come into contact with, otherwise known as contact tracing (**?**, 13).

It is not clear whether "the government asking you" means making the use of a COVID-19 app mandatory—a practice currently used in other countries—but some respondents could interpret the query as such.

As discussed in the main text, our study relied on a question that described the app using plain language, and we avoided to prime the merits versus the risks. To assess the sensitivity of our results to question wording, we randomly assigned respondents to one of three variants of the same question. The text appears in the next section. These variations in question wording, however, had no significant impact on the response.

In the next section we also report the question wording for the variables used in the main text. For the "Not Serious Enough" and "Worried" variables, we relied on the phrasing used in COVID-19 surveys from

the Washington Post and the Center for Democracy and Civic Engagement at the University of Maryland, to allow comparisons with existing datasets. For the purpose of our analysis, these variables were recoded to equal 1 for respondents who answered "Not seriously enough" and "Very worried", respectively, and 0 otherwise. Using the multinomial version of these variables did not affect the substantive conclusions reported in the text. The "Trudeau Approval" variable is coded 1 for respondent who believe the federal government did an excellent job handling the pandemic, 0 otherwise. The "Lost Job" variable equals 1 for respondents who reported having lost their job during the pandemic, and 0 otherwise.

Survey Questionnaire

[Not Serious] "During the COVID-19 lockdown, do you think most people have taken social distancing measures too seriously, not seriously enough, or are most people striking the right balance?"

- "Not taking seriously enough"
- "Striking the right balance"
- "Taking too seriously"

[Worried] "How worried, if at all, are you about close family members or friends becoming infected and seriously ill from the coronavirus?"

- "Very worried"
- "Somewhat worried"
- "Not worried"

[COVID Apps (Base Wording)] "Many COVID-19 apps are being used around the world to notify people who were in contact with someone infected (contact tracing apps). These apps record the interactions between users by detecting when two cell phones are close to each other.
These apps require the participation of health agencies to confirm who tested positive for COVID-19. Do you support the government's participation in a COVID-19 contact tracing app?"

- "Yes"
- "Yes, but only if using the app is voluntary"
- "No"

[COVID Apps (Health Agency Wording)] "Many COVID-19 apps are being used around the world to notify people who were in contact with someone infected (contact tracing apps). These apps record the interactions between users by detecting when two cell phones are close to each other.
These apps require the participation of health agencies to confirm who tested positive for COVID-19. In

most cases, COVID-19 apps are designed to notify health agencies when someone was in contact with an infected individual.
Do you support the government's participation in a COVID-19 contact tracing app?"

- "Yes"
- "Yes, but only if using the app is voluntary"
- "No"

[COVID Apps (Dilemma Wording)] "Many COVID-19 apps are being used around the world to notify people who were in contact with someone infected (contact tracing apps). These apps record the interactions between users by detecting when two cell phones are close to each other.
These apps require the participation of health agencies to confirm who tested positive for COVID-19. Some people claim that COVID-19 apps may pose a risk to fundamental rights, such as the right to privacy. Others claim these apps are needed to help reopen the economy while protecting public health. Do you support the government's participation in a COVID-19 contact tracing app?"

- "Yes"
- "Yes, but only if using the app is voluntary"
- "No"

[Open-Ended] "We would like to understand public opinion about COVID-19 apps. Could you please give us the main reason for your previous answer, in one or two sentences, using the box below."

[Trudeau Approval] "How would you rate the Canadian government's overall response to the coronavirus outbreak?"

- "Excellent"
- "Good"
- "Not so good"
- "Poor"

[Lost Job.] "Have you lost your job due to the COVID-19 lockdown?"

- "Yes"
- "No, but my hours were reduced"
- "No"
- "Not applicable/I did not work/I am retired"

Extended Results

Table A2 reports the full results for the logistic regression models used to calculate the differences in predicted probabilities reported in Figure 3 of the main text. The differences in predicted probabilities are averaged across all respondents after leaving all other covariates at their observed values. While the trend in experimental research is to model average treatment effects with linear regression, we should point out that the conclusions are the same using linear probability models.

Table A3 shows the full models used to create Figure 4 in the main text, where the outcome variables are two types of argument mentioned in the answers to the open-ended question on COVID-19 cell phone contact tracing apps. Once again, we used logistic regression models and the figure in the main text reports differences in predicted probabilities for a change from 0 to 1 in each independent variable, leaving other variables in the sample at their observed values. The effect sizes are virtually the same as coefficients from linear regression models. Note that the treatment effects on the other argument types are not statistically significant.

Finally, we report alternative specifications of the main models used to measure treatment effects in Figure 3. Table A4 shows the output from regression models without covariates, as well as a specification including an indicator of general support for "tougher" policies, which is an additive composite of four other survey questions asking respondents whether they support 1) mandatory facial masks in public transit, 2) the use of infrared cameras in public spaces, 3) mandatory COVID-19 testing in the workplace, and 4) stronger fines for people violating quarantine rules. In all cases, the dependent variable is the binary indicator of unconditional support for COVID-19 apps. These alternative specifications produce results that are consistent with those reported in the main text, although the effect of the "Not Serious Enough" covariate is not statistically significant.

Table A2: Explaining Support for COVID-19 Cell Phone Contact Tracing (Full Results)

	COVID-19 Contact Tracing Apps = Yes		
	Unweighted	Weighted	Wording Control
	(1)	(2)	(3)
Non-Compliers	0.388*	0.425**	0.429**
	(0.156)	(0.159)	(0.160)
Large Infection Rate	−0.022	0.027	0.027
	(0.154)	(0.158)	(0.158)
Dilemma Wording			0.066
			(0.155)
Health Agency Wording			−0.113
			(0.158)
Not Serious Enough	0.385**	0.383**	0.379**
	(0.129)	(0.132)	(0.132)
Worried	0.859***	0.903***	0.902***
	(0.131)	(0.134)	(0.134)
Trudeau Approval	0.860***	0.824***	0.819***
	(0.169)	(0.171)	(0.171)
Lost Job	0.126	0.057	0.049
	(0.169)	(0.176)	(0.177)
Above 56 Years Old	0.197	0.204	0.205
	(0.135)	(0.138)	(0.138)
Female	−0.179	−0.207	−0.211
	(0.126)	(0.129)	(0.129)
Atlantic (Base = Ontario)	−0.528*	−0.556*	−0.551*
	(0.264)	(0.271)	(0.272)
British Columbia (Base = Ontario)	−0.169	−0.115	−0.111
	(0.202)	(0.208)	(0.209)
Prairies (Base = Ontario)	−0.050	−0.054	−0.046
	(0.177)	(0.181)	(0.181)
Québec (Base = Ontario)	0.178	0.223	0.224
	(0.166)	(0.167)	(0.167)
Constant	−1.237***	−1.264***	−1.247***
	(0.181)	(0.185)	(0.204)
Observations	1,200	1,200	1,200

Notes: The table shows the full output of logistic regressions used to compute the differences in predicted probabilities reported in Figure 3 of the main text. The dependent variable equals 1 if the respondent supports COVID apps unconditionally, and 0 otherwise. The first model is unweighted. The last two models are computed using raking weights for interlocking quotas by age, gender and region. *p<0.05; **p<0.01; ***p<0.001

Table A3: Determinants of Arguments on COVID-19 Apps (Full Results)

	Others as a Threat	With Conditions
	(1)	(2)
Non-Compliers	0.512^{*}	0.101
	(0.221)	(0.191)
Large Infection Rate	−0.137	0.364^{*}
	(0.235)	(0.180)
Not Serious Enough	1.088^{***}	−0.138
	(0.192)	(0.157)
Worried	0.712^{***}	-0.784^{***}
	(0.186)	(0.176)
Trudeau Approval	0.138	−0.286
	(0.236)	(0.218)
Lost Job	−0.225	0.227
	(0.264)	(0.199)
Above 56 Years Old	−0.084	0.114
	(0.198)	(0.158)
Female	0.727^{***}	0.375^{*}
	(0.189)	(0.150)
Atlantic (Base = Ontario)	−0.708	0.464
	(0.369)	(0.269)
British Columbia (Base = Ontario)	-0.818^{*}	0.039
	(0.323)	(0.231)
Prairies (Base = Ontario)	-0.604^{*}	0.161
	(0.266)	(0.212)
Québec (Base = Ontario)	−0.432	−0.260
	(0.246)	(0.205)
Constant	-2.940^{***}	-1.437^{***}
	(0.271)	(0.216)
Observations	1,200	1,200

Notes: The table shows the full output of logistic regressions used to compute the differences in predicted probabilities reported in Figure 4 of the main text. The dependent variable equals 1 if the respondent invoked the argument indicated in the column header, and 0 otherwise. The models are computed using raking weights for interlocking quotas by age, gender and region. $^{*}p<0.05$; $^{**}p<0.01$; $^{***}p<0.001$

Table A4: Alternative Specifications (Treatment Effects)

	Linear	Linear	Logistic	Logistic
	(1)	(2)	(3)	(4)
Non-Compliers	0.076*	0.098**	0.317*	0.467**
	(0.036)	(0.033)	(0.151)	(0.167)
Large Infection Rate	−0.0004	0.016	−0.002	0.057
	(0.035)	(0.032)	(0.151)	(0.165)
Dilemma Wording		0.011		0.049
		(0.032)		(0.162)
Health Agency		−0.021		−0.126
		(0.032)		(0.166)
Tougher Policy Support		0.109***		0.637***
		(0.010)		(0.078)
Not Serious Enough		0.030		0.161
		(0.029)		(0.139)
Worried		0.161***		0.742***
		(0.031)		(0.141)
Trudeau Approval		0.151***		0.706***
		(0.038)		(0.177)
Lost Job		−0.012		−0.073
		(0.037)		(0.188)
Above 56 Years Old		−0.009		−0.051
		(0.029)		(0.140)
Female		−0.048		−0.247
		(0.026)		(0.135)
Atlantic (Base = Ontario)		−0.106*		−0.509
		(0.050)		(0.280)
British Columbia (Base = Ontario)		−0.015		−0.058
		(0.043)		(0.224)
Prairies (Base = Ontario)		0.030		0.147
		(0.038)		(0.193)
Québec (Base = Ontario)		0.058		0.288
		(0.035)		(0.174)
Constant	0.355***	−0.063	−0.598***	−3.063***
	(0.025)	(0.045)	(0.109)	(0.315)
Observations	1,200	1,200	1,200	1,200

Notes: Alternative specifications using the unconditional support for COVID-19 apps as the binary outcome variable. All models are computed using raking weights. *p<0.05; **p<0.01; ***p<0.001

Content Analysis

In this section, we report the coding scheme used to annotate the written answers to the open-ended question on COVID-19 contact tracing applications. The comments were annotated independently by three coders.

The coding scheme was created a priori (i.e. before commencing the manual annotation of comments) and is based on theory. The arguments against COVID-19 apps (concerns for privacy, the need to impose conditions, doubts about the effectiveness, and dismissal of the threat) were all part of the public debate and prominent in the literature cited in the main text. The arguments in favour of COVID-19 apps were selected according to our theory. The three categories are meant to measure whether the respondent's considerations are oriented toward society ("Societal Concerns"), the threat posed by others ("Others as a Threat") or the application itself ("App is Effective"). In particular, the second category is the mechanism expected in the disease avoidance hypothesis, whereby individuals come to support the app because of the threat posed by other groups, either the infected themselves or people whose behaviour pose a risk of contagion.

Our coding scheme originally included a separate category for supportive arguments evoking economic concerns—the need for a contact tracing app to help reopen the economy. However, that argument was seldom evoked by respondents, and we ultimately merged it with the "Societal Concerns" category for simplicity of presentation. The third coder used the coding scheme with seven substantive categories from the start.

The coding scheme below corresponds to the document used as instructions for the classification of comments. Each category is binary. The classification is not mutually exclusive: for each comment, we indicate if an argument is present or not. As a result, some comments may include more than one of the arguments from the coding scheme. Some respondents also expressed their ambivalence and explicitly mentioned arguments for and against the use of COVID apps.

Coding Scheme

What are the considerations/arguments emphasized by the respondent to explain their position regarding cell phone contact tracing apps? For each comment, check all categories that apply.

1. Risk for privacy; impact on civil liberties
   e.g. the app infringes on privacy; app violates civil liberties; government may use the data to track people
   ("Civil Liberties" in Table 1)

2. The app must be restricted in scope; conditions must be in place
   e.g. the app must be voluntary; only a part of the population should be required to use it;

government must guarantee the app won't be used after the pandemic
("With Conditions" in Table 1)

3. The app will not work
   e.g. not everyone has a phone; people may just stop carrying their phone; other methods are more useful
   ("Not Going to Work" in Table 1)

4. The threat isn't real
   e.g. the media/government exaggerated the threat of the virus; COVID is a conspiracy
   ("Threat Is Not Real" in Table 1)

5. Social benefits (societal considerations)
   e.g. we must protect the vulnerable; we need to stop the virus; we need to reopen the economy; public health is more important than anything else
   ("Societal Concerns" in Table 1)

6. Other people are a source of risk (focusing on others as a threat)
   e.g. people not following the rules pose a threat; app is needed because people won't quarantine; we need to know where infected people are; we need to avoid hot spots
   ("Others as a Threat" in Table 1)

7. The app is useful (considerations focusing on the app itself)
   e.g. it's an effective technology, the app will give useful information, the app worked well in other countries
   ("App is Effective" in Table 1)

8. Not applicable (other arguments)
   When the text makes another type of argument that does not fit any of the categories.

9. No response
   Non-response (blank text box) or gibberish comment.

The three human coders annotated all English language comments (978) and the average Cohen's Kappa coefficients (Cohen, 1960) are calculated on that common sample. The final categories used for analysis are based on the majority choice for English language comments. Two out of the three coders annotated the French language comments; the few cases of disagreements were resolved manually by discussion among the two coders. To further assess the robustness of our results, we replicated the analysis reported in the main text using a unanimity rule for English language comments (i.e. an

argument is considered to be present in the written answer only if all three coders agree). The findings remain consistent to those reported in the text when using the unanimity rule (in fact, the confidence in our inferences improves slightly when using unanimity).

References

Breton, Charles, Fred Cutler, Sarah Lachance, and Alex Mierke-Zatwarnicki. 2017. "Telephone versus Online Survey Modes for Election Studies: Comparing Canadian Public Opinion and Vote Choice in the 2015 Federal Election." *Canadian Journal of Political Science* 50(4): 1005–1036.

Cohen, Jacob. 1960. "A Coefficient of Agreement for Nominal Scales." *Educational and Psychological Measurement* 20(1): 37–46.

Coppock, Alexander, and Oliver A McClellan. 2019. "Validating the demographic, political, psychological, and experimental results obtained from a new source of online survey respondents." *Research & Politics* 6(1): 1–14.

Mainstreet Research. "Survey of Canada." Canada iPolitics, May 11, 2020. https://ipolitics.ca/wp-content/uploads/2020/05/Canada-iPolitics-11May2020.pdf.

Miratrix, Luke W, Jasjeet S Sekhon, Alexander G Theodoridis, and Luis F Campos. 2018. "Worth weighting? How to think about and use weights in survey experiments." *Political Analysis* 26(3): 275–291.

Moodie, Rosemary, Donna Dasko, Colin Deacon, Peter Loewen, and David TS. Fraser. 2020. "Canadian Public Support For The Use Of Mobile Phone Data To Fight The Covid-19 Pandemic." Ottawa: Senate of Canada. May 7, 2020.
